# Corrigendum: Genomic characterization of Haemophilus influenzae harbouring an exogenous resistance gene

**DOI:** 10.1099/jmm.0.002075

**Published:** 2025-09-16

**Authors:** Emi Tanaka, Takeaki Wajima, Sonoe Hirano, Shoji Seyama, Hidemasa Nakaminami, Kei-ichi Uchiya

**Affiliations:** 1 Department of Microbiology, Faculty of Pharmacy, Meijo University, Nagoya, Japan; 2 Department of Clinical Microbiology, School of Pharmacy, Tokyo University of Pharmacy and Life Sciences, Tokyo, Japan; 3 Department of Infectious Disease, Keio University Hospital, Tokyo, Japan

In the article titled ‘Genomic characterization of *Haemophilus influenzae* harbouring an exogenous resistance gene’ [1], incomplete accreditation was given to reference 15 (Johannessen *et al*. [2]) in several places within the article.

The following sections of the article are amended below to address this.

## Results (final paragraph)

A comparative analysis of the ICE region by Johannessen *et a*l. [15] suggested that Hi-228 (Tn*7100*) was derived from Hin1056 (ICE type I), followed by the insertion of Tn3 and Tn*7471* containing *mef* (*A/E*) (Fig. 2b). NRCH180136 was derived from 6P32H1 (ICE type II) by the insertion of Tn3 and Tn*7471* containing *mef* (*A/E*) (Fig. 2c). 2017-Y3 and 2014–102 arose by the insertion of a larger region of Tn*7471* (Fig. 2c). As described by Johannessen *et al*. [15]*,* Tn*7471* was similar to Tn*6822*, which is commonly found in *Streptococcus* (Fig. S4), suggesting that all *mef* (*A/E*)-positive strains obtained *Tn* on ICE from streptococci in the same niche.

## Discussion (third paragraph)

In addition, a strain (2017-Y3) with *mef* (*A/E*) and *tet* (*M*) on the ICE-harbouring *bla*_TEM-1_ was reported [14]. *H. influenzae* with *mef* (*A/E*) is very rare in Japan and worldwide [14, 17]. However, recently, these strains were isolated in Norway and Belgium [15, 16], suggesting that strains harbouring *mef* (*A/E*) might emerge worldwide. A comparative genomic analysis of these strains conducted by Johannessen *et al* [15] revealed that *mef* (*A/E*)-harbouring strains evolved from various types of ICEs through the serial acquisition of transposons (Fig. 3), and these *mef* (*A/E*) regions might be derived from *Streptococcus* Tn*6822* [15, 34], suggesting that they emerged by transfer from *Streptococcus* within similar niches. Since transposable genes were also present around *mef* (*A/E*), these elements may transpose to other genomic regions.

## Figure 2b caption

Fig. 2. Comparison of ICE domains. Results of the comparative genomic analysis were visualized using EasyFig. Purple and pink bands indicate forward and reverse matches, respectively. Each coding sequence is indicated by coloured arrows. (a) Comparison between Hin1056 (ICE type I) and 6P32H1 (ICE type II). (b) Comparison of ICE type I. Adapted from Fig. 1 in Johannessen *et al.* [15]. (c) Comparison of ICE type II.

## Figure 3 caption

Fig. 3. Image of ICE evolution. This figure shows the image of ICE evolution. The insertion of transposon is indicated by black straight arrows. The development of Tn*7100* is described in Johannessen *et al.* [15], and is represented visually here.

## Supplementary figure 4 caption

Fig. S4 Comparison of Tn*6822* and Tn*7471* Comparative genomic analysis results were visualised using EasyFig. Purple and pink bands indicate forward and reverse matches, respectively. Coding sequences are indicated by coloured arrows. Adapted from Fig. 3 in Johannessen *et al.* [15].

## Abbreviations

ICE: integrative and conjugative element
